# Dust Loading Performance of a Novel Submicro-Fiber Composite Filter Medium for Engine

**DOI:** 10.3390/ma11102038

**Published:** 2018-10-19

**Authors:** Jin Long, Min Tang, Zhaoxia Sun, Yun Liang, Jian Hu

**Affiliations:** 1 State Key Laboratory of Pulp and Paper Engineering, South China University of Technology, Guangzhou 510641, China; lj_king@139.com (J.L.); careysun988@163.com (Z.S.); liangyun@scut.edu.cn (Y.L.); ppjhu@scut.edu.cn (J.H.); 2 Filtration and Wet Nonwoven Composite Materials Engineering Research Center of Guangdong Province, Guangzhou 510641, China

**Keywords:** submicro-fiber, airborne dust, engine filtration, loading performance

## Abstract

Airborne dust can cause engine wear and contribute to engine gas emission. This study developed a novel submicro-fiber filter medium to provide protection to engines against dust. The wet-laid submicro-fiber medium was prepared by a dual-layer paper machine, and its dust loading performance was compared with other filter media during laboratory and field tests. During the laboratory tests, the dust holding capacity of the wet-laid submicro-fiber medium was 48% and 10% higher than that of the standard heavy-duty medium and electrospun submicro-fiber medium, respectively. During the field tests, the pressure drop of the wet-laid submicro-fiber filter was 45% lower than that of the standard heavy-duty filter after 10,000 km of operation. It was found that there were two crucial ways to design a better filter medium for protection against dust. Firstly, the surface loading rather than the depth loading was preferred for dust filtration. The submicro-fiber layer kept large amounts of dust particles from penetrating into the depth of filter medium. Secondly, particles were captured preferably by fibers rather than pores. The unique fibrous structure of the wet-laid submicro-fiber medium made more particle deposition take place on fibers via interception and inertial impaction.

## 1. Introduction

Arid and semi-arid areas, which cover approximately one-third of land surfaces, are a major source of mineral dust [1]. If meteorological conditions are favorable, the dust is mobilized and further emitted into the atmosphere. Besides natural processes, a significant amount of dust contaminants are generated by anthropogenic activities, such as farming, industrial manufacturing and mining operations [2]. For motor vehicles, dust suspended in the air is hazardous and can degrade vehicle performance because the combustion engine of the vehicle is exposed to large quantities of air. Airborne dust (quartz) is highly abrasive and is the most common cause of high wear of critical parts, such as piston rings and cylinder walls in engines [3]. Studies have shown that engine life is dependent upon the cleanliness of the air taken in [4]. Engine wear is produced by particles in the size range of 1–40 µm, where the most harmful particles are in the range of 1–20 µm [5]. Dust particles may also significantly contribute to the total engine emissions, including crankcase emissions [6]. According to Schilling [7], 30% of contaminants entering the engine can pass out of the exhaust.

Air filtration is applied to provide protection against the effects of contaminated air. The effects of the engine intake air filter can be both positive and negative [8]. The positive side is to prevent the decrease in engine performance, however, placing an air filter in the path of air coming into the engine can cause a pressure drop, leading to extra energy costs and reduced engine performance. The pressure drop is proportional to the energy consumption of the air filter, according to Eurovent 4/21 (2014) [9]. The challenge is to achieve a lower pressure drop and longer filter lifetime while removing a satisfactory number of particles.

In the combustion engine industry, fibrous filter media are used to prevent airborne particles from entering the engine. Cellulose-type filter media, made of relatively large fibers with fiber diameters, usually larger than 10 microns, are often used in engine air filtration. To improve filtration performance, the application of ultrafine fibers has become of interest in academic research and within the industry [10,11,12,13]. Many theoretical calculations and experimental results of submicro-fiber filter medium have shown that the filtration efficiency could be dramatically improved by the gas slip effect and a large specific surface area [14]. Recently, nanofiber sponge or aerogel were introduced as new porous materials for filtration [15,16]. A popular method of producing submicro-fiber composite filter medium is adding a submicro-fiber layer via electrospinning [17,18,19,20]. Donaldson Company (Bloomington, MN, USA) had successfully introduced electrospinning submicro-fiber technology in industrial applications in 1981. However, there are still many challenges, such as high cost, submicro-fiber layer adhesion, and uniformity, and hazards associated with solvent removal and disposal [6]. The mechanical strength of the thin electrospun submicro-fiber layer is poor and can be damaged during the transportation process [21].

The advantage of using a submicro-fiber provides a valid case for relatively clean filters. When dust deposits on submicro-fiber, the benefit of a low pressure drop diminishes with increasing amounts of deposited dust. For military vehicles in high dust concentration environments, a self-cleaning method can be used to blow off dust cake using a pulsejet and make the filter medium clean again [22]. However, most civilian vehicles are not equipped with self-cleaning devices, which means that the air filter would capture dust particles constantly without dust cake removal. There has been a great deal of effort towardsperformance evaluation of a clean submicro-fiber composite without aerosol loading, including submicro-fiber/microfiber mixture [23] and various stack-up nanocomposite [24,25]. Leung et al. [26] studied microfiber/submicro-fiber composites under nano-aerosol loading. However, very limited study has been conducted on submicro-fiber composites against coarse particles that are more harmful to the engine. It is important to design a submicro-fiber medium with a slowly increasing rate of pressure drop in the presence of a high concentration of dust.

To develop a new submicro-fiber medium, this study will combine the ideas of submicro-fiber and multilayer papermaking to laminate a submicro-fiber layer on the substrate via the wet-laid process. During the wet-laid process, resin impregnation for a submicro-fiber layer can be easily applied, which will result in a robust submicro-fiber layer and strong adhesion with a substrate. Langner and Greiner [27] discussed the application of electrospun ultrafine fiber in wet-laid process for filtration. The wet-laid process is also able to use submicro-fibers with superior filtration performance, such as glass wool fiber, which is the main component of cleanroom high efficiency particulate air filters [28]. This paper will present a new method to prepare composite filter medium with unique submicro-fiber layer via paper machine. The preparation of a submicro-fiber composite medium was conducted on an industrial paper machine. The loading performance and dust deposition behavior of the novel submicro-fiber medium were compared with a commercial standard heavy-duty medium and an electrospun submicro-fiber medium to provide a fundamental discussion on the submicro-fiber structure design against high dust concentrations.

## 2. Materials and Methods

### 2.1. Preparation of Wet-laid Submicro-Fiber Composite Filter Medium

The preparation of a composite filter medium was conducted on a pilot-scale customized paper machine in Fibrway Co., Ltd. (Guangzhou, China). The experimental results on the paper machine were more applicable for real applications compared to paper preparation in the laboratory [29]. During laboratory processes, water flows perpendicularly to a fixed forming screen, while for an industrial paper machine, water flows to a moving forming screen at a certain angle, which results in a different fiber orientation and structure.

**Figure 1:**
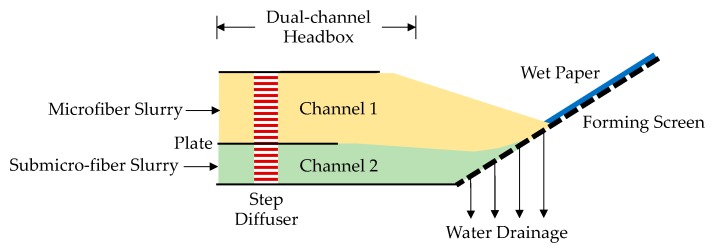
Schematic diagram of dual-channel headbox.

The main preparation processes included pulp preparation, sheet forming, drying, and coating. There were two sets of the pulp preparation system: Microfiber and submicro-fiber slurry. For the microfiber slurry, the fibers consisted of 20% softwood pulp (Weyerhaeuser Co., Seattle, WA, USA), 60% flash-dried hardwood pulp (Suzano, Sao Paulo, Brazil), 15% 1.4 D × 5 mm polyethylene terephthalate (PET, from Kuraray Co., Ltd., Tokyo, Japan) fiber, and 5% 3-µm glass fiber was dispersed in clean water and then stored in a tank with continuous agitation to keep fibers away from entanglement. The microfiber slurry with a pulp consistency of 0.1 wt.% was pumped to channel 1 of the headbox, as shown in Figure 1. For the submicro-fiber slurry, glass wool fibers (Dongxiang Glass Microfiber Co., Ltd., Shenyang, China) with an average diameter of 0.6 µm were dispersed in water with a pH of 2.5 [30] and then stored in a tank with continuous agitation. The submicro-fiber slurry with a consistency of 0.02 wt.% was pumped to channel 2 of the headbox, as shown in Figure 1. The mass ratio of the microfiber to submicro-fiber layer was 10:1.

This dual-channel headbox had been patented by the authors [31]. In the dual-channel headbox, fluids in both channels were stabilized by a step diffuser and then became a steady laminar flow. These two channels were separated by a plate so that the submicro-fiber slurry did not mix with the microfiber slurry. Before entering the water drainage section, the slurries in the two channels converged at the end of the plate and were kept separated without mixing due to the steady laminar flow. Water from the submicro-fiber slurry drained first, and then the submicro-fiber deposited on the forming screen. The forming screen was a multilayer wire of PET and nylon fiber strands with a top layer pore size of 100 µm. After the submicro-fiber layer formed, microfibers started to deposit on the submicro-fiber and the dual-layer paper sheet was formed. The crucial part of the drainage section was to keep the water drainage process steady and prevent the dual-layer structure from being damaged. To achieve this goal, the submicro-fiber layer was put underneath the microfiber layer because the submicro-fiber deposition on the forming screen can slow down the initial water-draining rate and thus, make the water drainage steadier.

**Figure 2:**
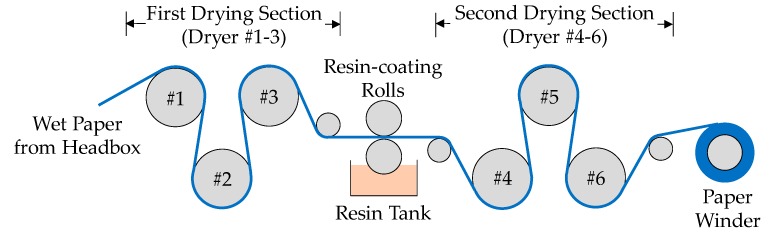
Schematic diagram of drying and coating process.

The wet paper sheet was then transferred to the drying and coating section, as shown in Figure 2. The dried paper from the first drying section was transferred to the gap of two resin-coating rolls. The lower roll brought 20 wt.% waterborne acrylic polymer (PR-36, Wuwei Co., Ltd., Guangzhou, China) from the resin tank to saturate the paper. This polymer can enhance the strength of the paper, especially the weak submicro-fiber layer. The second drying section was used to dry the coated paper. A major problem that existed during the drying of the coated porous material was that the resin could migrate from the inner part of paper to the surface when heated [32] and gradually clog the surface pores. Pore clogging can remarkably reduce the filtration performance and cancel out the advantages of the submicro-fiber. To mitigate binder migration, the temperature of each dryer in the second drying section was accurately controlled. The cylinder surface temperatures of dryer 4, 5, and 6 were 75 ± 5 °C, 105 ± 5 °C, and 125 ± 5 °C, respectively. The resin content in the final dried paper was 20%. After drying and winding, the preparation of the submicro-fiber medium was complete.

The running speed of the pilot-scale paper machine was 5 m/min. The basic weight of the filter paper was 120 ± 3 g/m^2^. The full width of the final paper sheet was 0.7 m after cutting 0.05 m on both edges. Filter paper samples for this study were taken 1 h after stable operation of the paper machine was achieved.

### 2.2. Loading Performance Evaluation in Laboratory—Filter Media Specifications

The loading performance of the wet-laid submicro-fiber composite medium was compared to the two other filter media. The standard heavy-duty filter medium (H&V Inc., East Walpole, MA, USA) is currently used in many inlet filters of engineering machinery. The electrospun composite medium (Fibrway Co., Ltd., Guangzhou, China) is known for its outstanding performance in many studies [19,20]. The submicro-fiber in the electrospun medium was made from nylon-66 (Elmarco, Liberec, Czech Republic). The basic properties of these filter media are described in Table 1. The micro-structure of these filter media was observed by scanning electron microscope (Phenom^TM^, Phenom-World B.V. Inc., Eindhoven, The Netherland). The pore size was measured by capillary flow porometer (model: CFP-1100A, Porous Materials Inc., Ithaca, NY, USA).

### 2.3. Loading Test Setup in Laboratory

**Figure 3:**
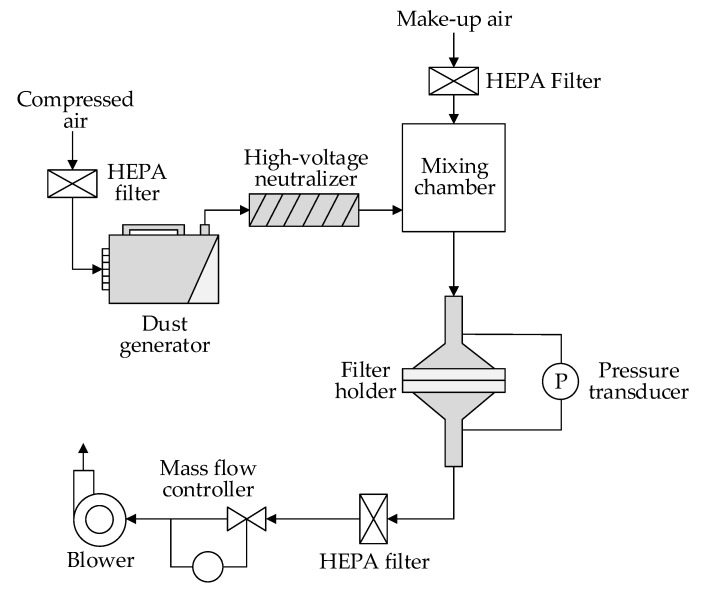
Schematic diagram of dust loading test setup.

The loading test rig (MFP 3000, Palas GmbH, Karlsruhe, Germany) was used to test the dust holding capacity according to the ISO 5011 [36] standard, as shown in Figure 3.

The particles used in the test were ISO 12103-1 [38] A2 fine dust (Powder Technology Inc., Arden Hills, MN, USA). The volume mean diameter of A2 fine dust was approximately 10 μm, which was suitable to evaluate the filter medium for the engine. When the loading test started, dry A2 dust was dispersed by compressed air (0.6 MPa), and then neutralized to achieve Boltzman charge equilibrium by an ion stream from a high-voltage neutralizer. Make-up air was mixed with particles in the mixing chamber to achieve a final particle mass concentration of 1.0 mg/m^3^. The filter medium clamped in the filter holder with a test area of 100 cm^2^ was challenged by the dust particles under a constant flow rate controlled by a mass flow controller. The testing face velocity was 11.1 cm/s, which was close to the operating velocity in real applications [39]. The test stopped after the pressure drop reached 2 kPa. During the test, the temperature was 21 °C and the relative humidity (RH) was 50 ± 10%. For every sample, the loading test was repeated 5 times.

The filter medium was weighed when it was clean (*M*_0_) and fully loaded (*M*_1_). The final dust mass deposit, *M_final_*, was calculated by Equation (1).

(1) $$M_{final}=\frac{\left(M_{1}-M_{0}\right)}{S}$$

where *S* is the test area of filter medium.

Because the gravimetric efficiency for all filter media was close to 100%, as shown in Table 1, it was considered that all particles were deposited on the filter media. Dust mass deposit per unit area, *M_i_*, at loading time of *t_i_* can be calculated by Equation (2).

(2) $$M_{i}=\frac{t_{i}}{t_{total}}\times M_{final}$$

where *t_total_* is the total loading time (min).

The curves of pressure drop versus dust mass deposit per unit area were used to analyze the loading performance of the different media. Two parameters obtained from the pressure drop curves were considered for discussion: Dust holding capacity and energy consumption. Dust holding capacity was defined as the dust mass deposit per unit area when the pressure drop reached 2 kPa. The energy consumption of different filter media under a fixed amount of dust mass deposit can be compared by calculation methods described in Eurovent 4/21 [9].

**Figure 4:**
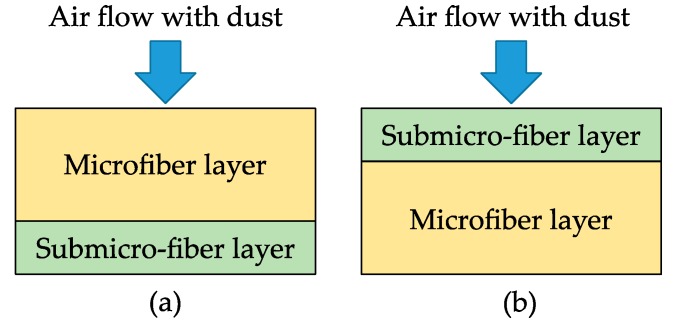
Loading tests of wet-laid submicro-fiber composite medium using: (**a**) microfiber layer as inlet and (**b**) submicro-fiber layer as inlet.

The loading performance of the wet-laid submicro-fiber composite medium and two commercial filter media was evaluated in the laboratory. Because the wet-laid submicro-fiber composite medium was a dual-layer structure, loading tests using the microfiber or submicro-fiber layer at the inlet were conducted to analyze the difference in filtration performance, as shown in Figure 4.

### 2.4. Loading Performance Evaluation in Field Test

**Figure 5:**
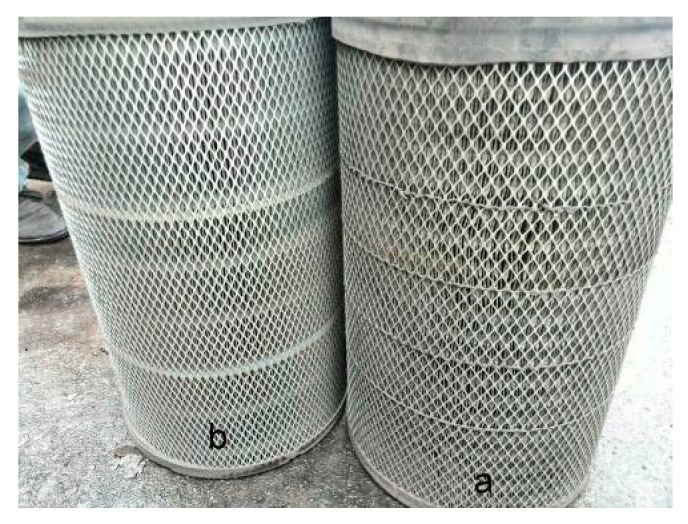
OEM inlet air filter (**a**) and new submicro-fiber filter (**b**).

The loading performance of the wet-laid submicro-fiber composite medium and the standard heavy-duty filter medium were evaluated in the field test. Because one sample of the electrospun composite medium was not big enough to make a full-size filter, no field test was conducted for the electrospun medium. During the field test, two heavy-duty trucks (Tianlong 375, Dongfeng Motor Corp., Wuhan, China) with close mileage were used. The original equipment manufacturer (OEM) air filter (Model: 3048u, Shanghai Fleetguard Filter Co., Ltd., Shanghai, China) for this truck was made by the standard heavy-duty filter medium. In comparison, a filter based on identical filter geometry was made using the wet-laid submicro-fiber medium, as shown in Figure 5.

**Figure 6:**
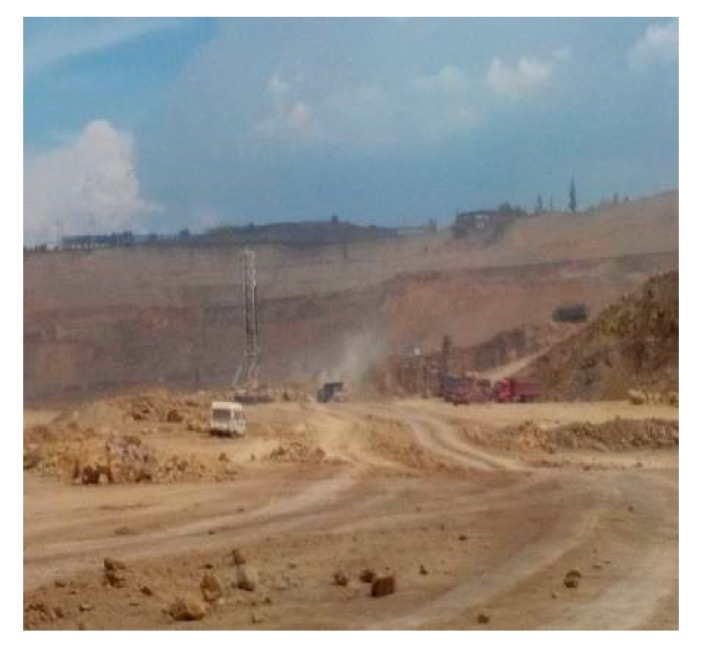
Dusty conditions of field test site.

The two trucks equipped with the OEM filter and submicro-fiber filter operated at the same coal mine (Lyuliang Coal Mine, Shanxi, China) under the same tasks. The surrounding region of this coal mine was semi-arid, and many roads are unpaved and dusty, as shown in Figure 6, which was a harsh environment for the air filter. The nominal flow rate of air intake filter in the truck was 1500 m^3^/h. After running 10,000 km, these two filters were taken out of the trucks and put into an ISO 5011 [36] test rig to conduct the pressure drop measurement under an airflow rate ranging from 750 to 1800 m^3^/h. The pressure drop results were used to evaluate the loading performance of the wet-laid submicro-fiber medium and the standard heavy-duty filter medium.

## 3. Results and Discussion

### 3.1. Properties and Structural Analysis of Wet-Laid Submicro-Fiber Medium

The properties of the wet-laid submicro-fiber medium are in Table 1. The wet-laid submicro-fiber medium had a close basic weight and gravimetric efficiency compared to the other two media. The initial pressure drop of the wet-laid submicro-fiber medium was 29% lower than that of the standard heavy-duty medium, but 44% higher than that of the electrospun medium. Although the initial pressure drop can indicate initial performance, it did not represent the overall performance of the loading, which is discussed in the following sections. The bending stiffness in the machine direction, which indicated the ability of sustaining the pleat structure in the operation, was very close for the three different filter media.

**Figure 7:**
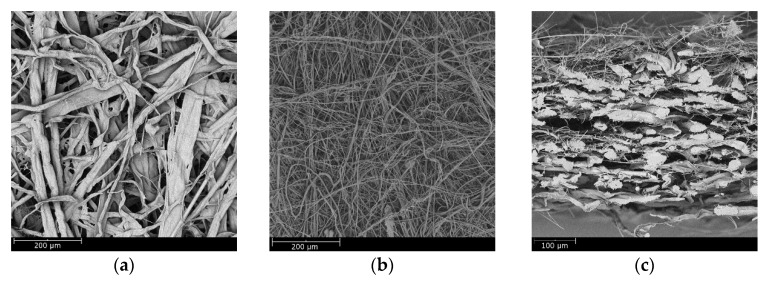
SEM images of wet-laid submicro-fiber medium: (**a**) microfiber layer, (**b**) submicro-fiber layer, and (**c**) cross-section.

Figure 7 shows scanning electron microscopy (SEM) images of the wet-laid submicro-fiber medium. The microfiber and submicro-fiber layer were bonded together to create a dual-layer structure. Due to the intrinsic stiffness of glass, the glass wool fiber in the submicro-fiber layer was relatively straight and stiff, thus sustaining a highly porous fiber network. Few pores were clogged by resin in either the submicro-fiber or microfiber layer, which indicated that the temperature profile of the second drying section was an effective strategy to mitigate binder migration.

### 3.2. Loading Performance of Wet-laid Submicro-Fiber Medium in Laboratory

**Figure 8:**
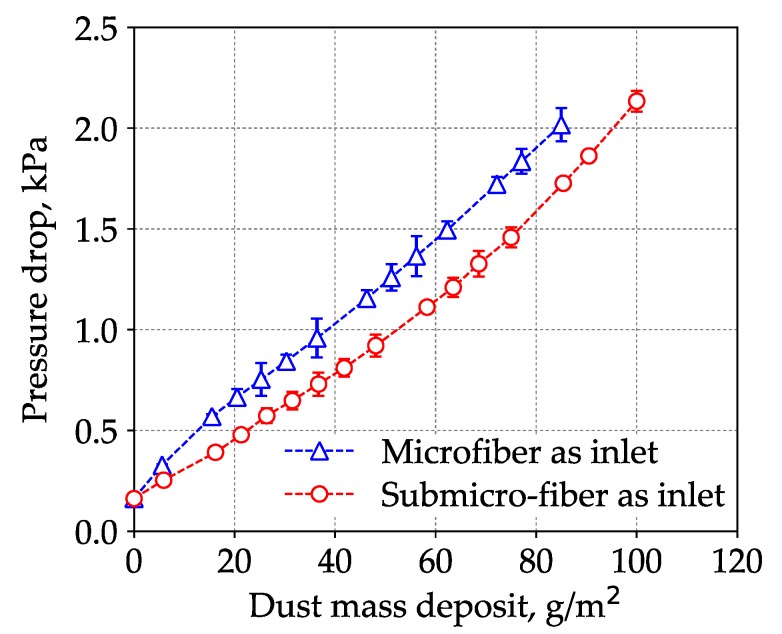
Loading results of wet-laid submicro-fiber medium with the microfiber or submicro-fiber layer as inlet.

When a dual-layer filter medium was used for filtration, it was important to determine which media was the inlet. Several researchers have studied the loading difference of the microfiber and submicro-fiber layers. Leung and Hung [40], Leung et al. [41], and Tang et al. [42] predicted that because the surface loading was taking place on the submicro-fiber layer, the pressure drop across the submicro-fiber filter could rise at a much faster rate than the microfiber filter. It was also hypothesized that the microfiber layer could be placed upstream in a dual-layer filter to help collect parts of the particles, thus suppressing the increased rate of pressure drop. However, their studies focused on fine particles (e.g., PM_2.5_), and it must be noted that the loading behavior of coarse particles was very different. Figure 8 shows the loading results of the wet-laid submicro-fiber medium with the microfiber or submicro-fiber layer as the inlet.

The loading results demonstrated that when the submicro-fiber layer was placed as the inlet, the pressure drop increased at a lower rate. When the microfiber layer was the inlet, the dust holding capacity was 84 ± 2 g/m^2^. When the submicro-fiber layer was the inlet, the dust holding capacity was 95 ± 1 g/m^2^, which was 13% higher than that of the microfiber layer as the inlet. This meant that when the filter medium was challenged by dust particles, the lifetime was 13% longer if the submicro-fiber layer was placed in the upstream. This conclusion was contradictory to the conclusion of Leung et al. [41], which indicated that the loading behavior of coarse particles was different from that of fine particles.

**Figure 9:**
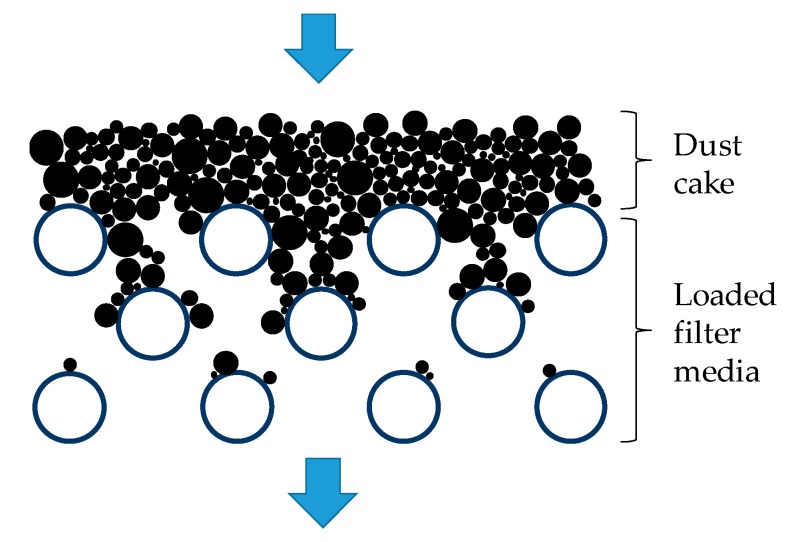
Schematic of dust cake and loaded filter medium.

The pressure drop of the loaded filter medium can be considered as the sum of the pressure drop across the loaded filter medium and the pressure drop of the dust cake, as shown in Figure 9. 

According to Endo et al. [43,44], the pressure drop of dust cake can be calculated by Equation (3). It can be seen from Equation (3) that the pressure drop of dust cake was related to the properties of fluid and dust, and independent of filter medium. The latter part of the two curves in Figure 8 showed a similar increasing rate, which demonstrated that the pressure drop of the dust cake had no dependency on the filter medium. Both theoretical model and experimental results indicated that it was the particles’ deposition pattern inside the filter medium that determined the pressure drop evolution in the loading. Therefore, the interaction between the particles and the fibrous structure in the early loading stage was the key to analyzing the results in Figure 8.

(3) $$\Delta P_{cake}=180\mu u_{s}H\frac{{\left(1-\varepsilon \right)}^{2}}{\varepsilon^{3}}\frac{\kappa }{d_{vg}^{2}\exp(4\ln^{2}\sigma_{g})}$$

where Δ*P_cake_* is pressure drop across the dust cake; *μ* is gas viscosity; *u_s_* is face velocity; *H* is cake height; *ε* is cake porosity; *κ* is dynamic shape factor of A2 dust; *d_vg_* is geometric mean of volume equivalent diameter; *σ_g_* is geometric standard deviation.

**Figure 10:**
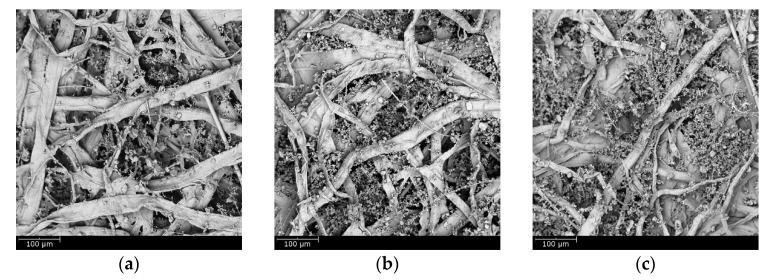
SEM images of dust deposition when microfiber layer was placed as inlet and pressure drop increase by: (**a**) 10 Pa, (**b**) 50 Pa, and (**c**) 100 Pa.

**Figure 11:**
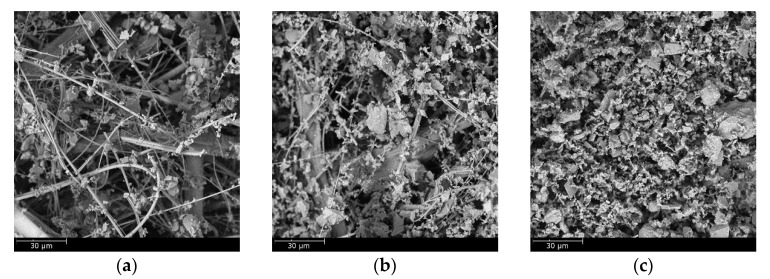
SEM images of dust deposition when submicro-fiber layer was placed as inlet and pressure drop increase by: (**a**) 10 Pa, (**b**) 50 Pa, and (**c**) 100 Pa.

To illustrate the loading behavior of the particles, SEM images of dust deposition on the filter medium were obtained when the pressure drop increased 10, 50, and 100 Pa, respectively, as shown in Figure 10 and Figure 11. When the microfiber layer was placed as the inlet, dust particles penetrated the depth of the filter medium. The deposited particles bridged the pores and gradually clogged the pores. The dust particles captured inside the filter medium reduced the porosity and led to a higher pressure drop. When the submicro-fiber layer was placed as the inlet, less dust particles penetrated the depth of the filter medium and surface loading occurred rapidly. Based on the results in Figure 8, the filter medium clogged by dust particles was more difficult for air to permeate than the dust cake. Therefore, it was preferable for surface loading to happen earlier for coarse particles, while in the studies of Leung et al. [41] and Tang et al. [42], surface loading should take place later when fine particles were tested. For this reason, the submicro-fiber layer was placed as the inlet in the following discussions.

### 3.3. Loading Performance Comparison Between Wet-laid Submicro-Fiber Medium and Standard Heavy-duty Medium in the Laboratory and Field

**Figure 12:**
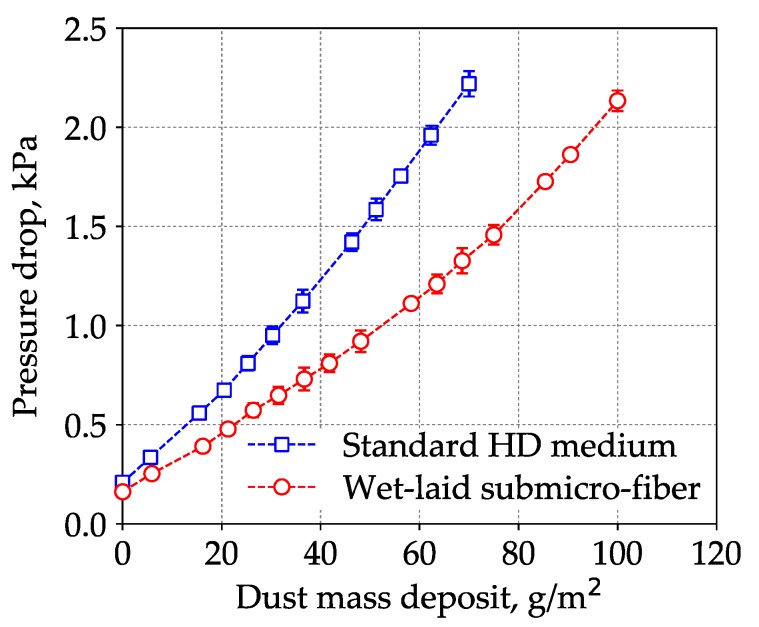
Pressure drop curves of standard heavy-duty (HD) medium and wet-laid submicro-fiber medium in the loading process.

The loading results of the wet-laid submicro-fiber medium and the standard heavy-duty medium are shown in Figure 12. The pressure drop of the standard heavy-duty medium increased faster than that of the wet-laid submicro-fiber medium. When the pressure drop reached 2 kPa, the dust mass deposit of the standard heavy-duty medium was 64 g/m^2^, which meant that the dust holding capacity of the wet-laid submicro-fiber medium was 48% higher than that of the standard heavy-duty medium.

The energy consumption of air filter media was proportional to average pressure drop in loading process [9]. Δ*p*(*M_i_*) was the pressure drop when dust mass deposit was *M_i_*. The average pressure drop in a complete loading test can be calculated by Equation (4). The polynomial of fourth order Δ*p*(*m*) = *a_n_M_i_*^4^ + *b_n_M_i_*^3^ + *c_n_M_i_*^2^ + *d_n_M_i_* + Δ*p_I_* and parameters *a_n_*, *b_n_*, *c_n_* and *d_n_* were used to curve fit the pressure drop data as a function of loading time. Δ*p_I_* is the initial pressure drop of filter media.

(4) $$\bar{\Delta p}=\frac{1}{M_{final}}\int_{0}^{M_{final}}\Delta p(M_{i})dM_{i}$$

Under a dust mass deposit of 64 ± 1 g/m^2^, the estimated energy consumption of the wet-laid submicro-fiber medium was 36% lower than that of standard heavy-duty medium based on calculation of average pressure drop via Equation (4). The laboratory results showed that the wet-laid submicro-fiber medium had a longer lifetime and lower energy consumption than the standard heavy-duty medium when challenged by dust particles.

**Figure 13:**
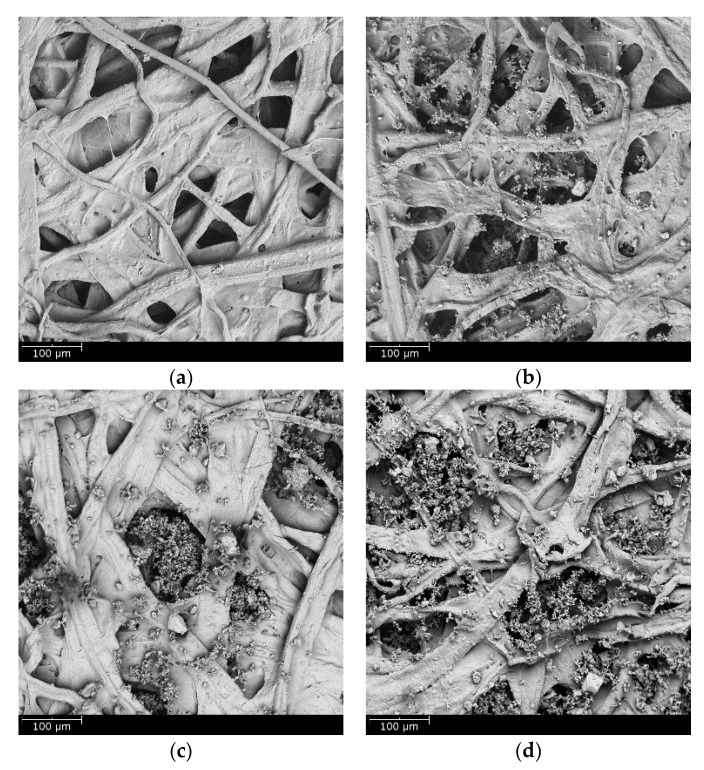
SEM images of dust deposition of standard heavy-duty filter medium: (**a**) clean medium, (**b**) pressure drop increase of 10 Pa, (**c**) pressure drop increase of 50 Pa, and (**d**) pressure drop increase of 100 Pa.

The SEM images of the clean standard heavy-duty medium and the loaded medium when the pressure drop increased 10, 50, and 100 Pa were obtained, respectively, as shown in Figure 13. The dust particles penetrated into the depth of the fibrous structure. Surface loading took place later than the wet-laid submicro-fiber medium compared to Figure 11. The dust loading behavior was similar to that of the microfiber layer in Figure 10. The results further confirmed that depth filtration was not suitable for coarse particle filtration.

**Figure 14:**
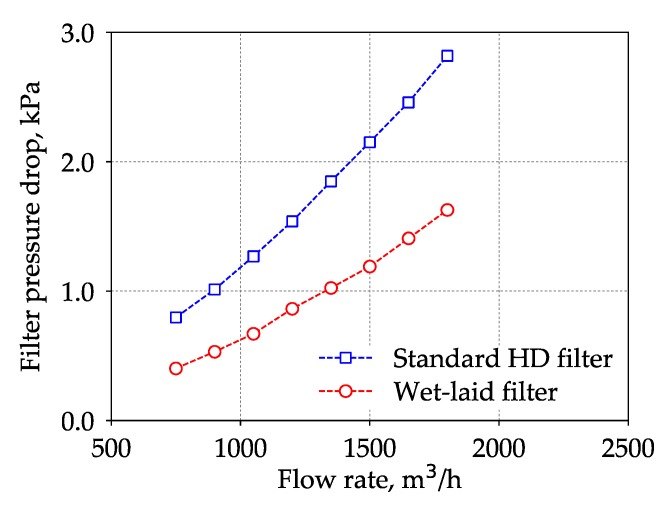
Pressure drop of used standard heavy-duty (HD) filter and wet-laid submicro-fiber filter under different flow rates after running 10,000 km.

Figure 14 shows the pressure drop of the standard heavy-duty filter and the wet-laid submicro-fiber filter under different flow rates after 10,000 km of operation. Under the nominal flow rate of the air filter, 1500 m^3^/h, the pressure drop of the standard heavy-duty filter was higher than 2 kPa and the filter service life ended. The pressure drop of the used wet-laid submicro-fiber filter was approximately 45% lower than that of the standard heavy-duty filter, which demonstrated the advantage of the novel filter medium in the field test. During the laboratory tests, when the pressure drop of the standard heavy-duty medium reached 2 kPa, the pressure drop of the wet-laid submicro-fiber medium under the same dust mass deposit was 38% lower than that of the standard heavy-duty medium, which was close to the field test results. The field test results showed that the wet-laid submicro-fiber medium had a longer lifetime when challenged by dust particles.

### 3.4. Loading Performance Comparison Between Wet-Laid Submicro-Fiber Medium and Electrospun Composite Medium in Laboratory

**Figure 15:**
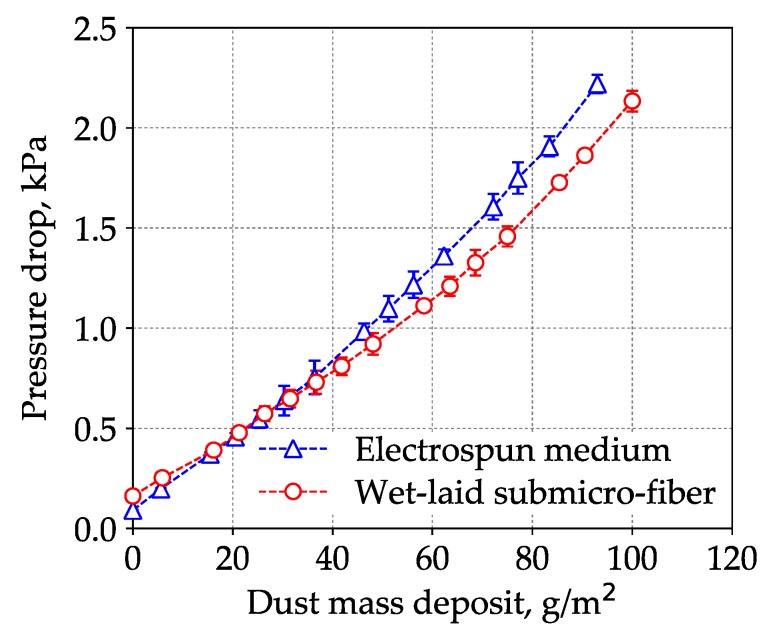
Pressure drop curves of electrospun composite medium and wet-laid submicro-fiber medium in the loading process with submicro-fiber as upstream.

The loading results of the wet-laid submicro-fiber medium and an electrospun composite medium are shown in Figure 15. The initial pressure drop of the electrospun medium was lower, but the pressure drop increased faster than the wet-laid submicro-fiber medium. When the pressure drop reached 2 kPa, the dust mass deposit of the electrospun medium was 86 ± 1 g/m^2^, which meant that the dust holding capacity of the wet-laid submicro-fiber medium was 10% higher than that of the electrospun medium. Under a dust mass deposit of 86 g/m^2^, the estimated energy consumption of the wet-laid submicro-fiber medium was 10% lower than that of the electrospun medium based on calculation of average pressure drop via Equation (4). The laboratory results showed that the wet-laid submicro-fiber medium had a longer lifetime and lower energy consumption than the electrospun medium when challenged by dust particles.

**Figure 16:**
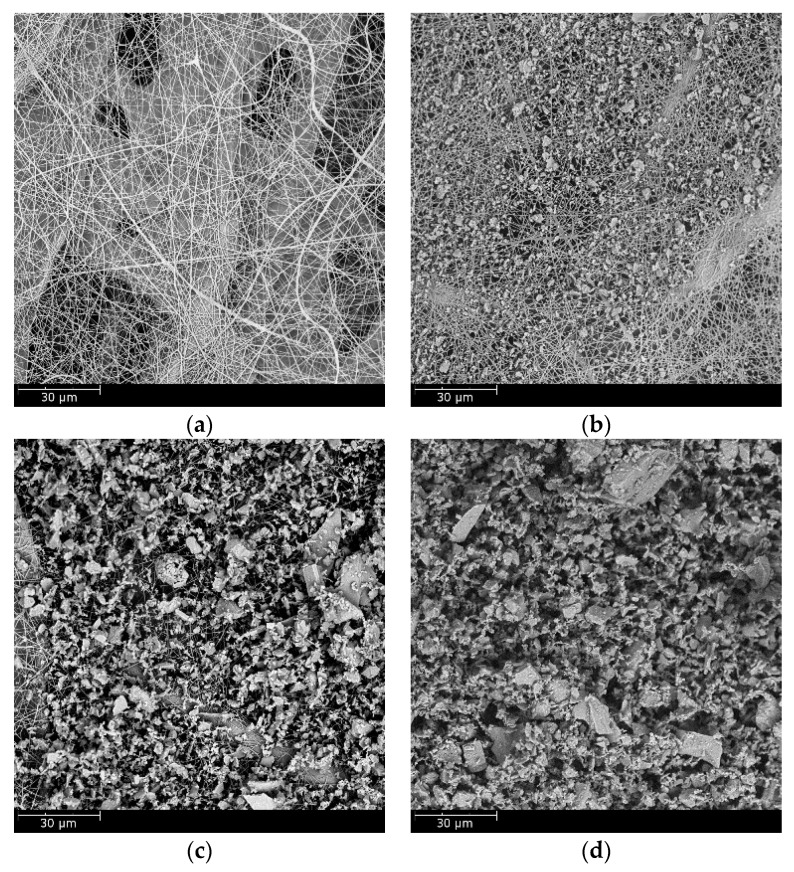
SEM images of dust deposition of electrospun composite medium: (**a**) clean medium, (**b**) pressure drop increase of 10 Pa, (**c**) pressure drop increase of 50 Pa, and (**d**) pressure drop increase of 100 Pa.

The SEM images of the clean electrospun composite medium and loaded filter medium when the pressure drop increased 10, 50, and 100 Pa were obtained, respectively, as shown in Figure 16. The electrospun medium was believed to be a typical surface loading material. When the pressure drop increased 10 kPa, the electrospun medium had already shown evident signs of surface loading. Although surface loading was preferred in the removal of coarse particles, the process of surface loading would affect the loading performance.

**Figure 17:**
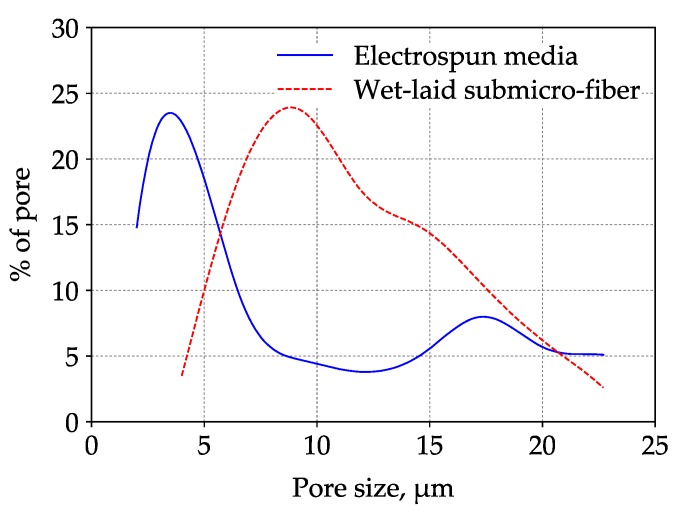
Pore size distribution of electrospun media and wet-laid submicro-fiber media.

The governing mechanisms of capturing coarse particles were interception, inertial impaction, and sieving [14]. Particles were captured by fibers due to interception and inertial impaction, while particles were arrested at the pores (smaller than the particle diameter) due to sieving. Figure 11 shows that the dust particles were largely captured by submicro-fibers of the wet-laid medium, while particles were largely arrested by pores of the electrospun medium as shown in Figure 16. Figure 17 was the pore size distribution of electrospun media and wet-laid submicro-fiber media. The peak pore size of electrospun media and wet-laid submicro-fiber media was 3.3 μm and 8.8 μm, respectively. The pore size distribution of electrospun media was narrower than that of wet-laid submicro-fiber media. Since the volume mean diameter of A2 fine dust was approximately 10 μm [38], more dust particles were captured by pores instead of fibers for electrospun media than wet-laid submicro-fiber media. When the pores were blocked by larger particles, the airflow path through the fibers and particles was restricted. Airflow velocity in the restricted pores rapidly increased and thus the pressure drop increased. The unique fibrous structure of the wet-laid submicro-fiber medium made more particle deposition take place on fibers via interception and inertial impaction rather than sieving, which led to a porous structure with a larger airflow path. Therefore, the wet-laid submicro-fiber medium showed a better loading performance against coarse particles than the electrospun medium.

The current study is limited to room temperature and controlled RH (50 ± 10%). In the future, the effect of excess RH and temperature on dust loading performance of wet-laid submicro-fiber media should be studied.

## 4. Conclusions

In this study, a novel wet-laid submicro-fiber composite medium was prepared by a dual-layer paper machine, and its loading performance and dust deposition behavior was compared with a standard heavy-duty medium and electrospun medium in laboratory and field tests. It was found that:

(1) The submicro-fiber layer, laminated by dual-channel headbox technology and reinforced by acrylic resin, was highly porous without pore clogging. The loading results demonstrated that when the submicro-fiber layer was placed as the inlet, the pressure drop increased at a slower rate than that when the microfiber layer was the inlet. It was concluded that it was preferable for surface loading to happen earlier for dust particles.

(2) During the laboratory test, the dust holding capacity of the wet-laid submicro-fiber medium was 48% higher than that of the standard heavy-duty medium, and the estimated energy consumption was 36% lower. In the field test, the pressure drop of the wet-laid submicro-fiber filter was approximately 45% lower than that of the standard heavy-duty filter after 10,000 km of operation. This showed that the wet-laid submicro-fiber medium had a longer lifetime and lower energy consumption than the standard heavy-duty medium when challenged by dust particles.

(3) The dust holding capacity of the wet-laid submicro-fiber medium was 10% higher than that of the electrospun medium, and the estimated energy consumption was 10% lower. The key point of submicro-fiber medium design was that the particles were preferable to be captured by fibers rather than pores. The fibrous structure of the wet-laid submicro-fiber medium made more particle deposition take place on fibers via interception and inertial impaction. Thus, in this work, the submicro-fiber medium showed a lower pressure drop than the electrospun medium in the loading process.

## Figures and Tables

**Table 1:** Physical properties of wet-laid composite medium and two other filter media.

Parameters	Test Data			Test Standard
	Wet-Laid Composite Medium	Standard Heavy-Duty Filter Medium	Electrospun Composite Medium	
Basis weight (g/m^2^)	119	110	118	TAPPI T410 [33]
Thickness (mm)	0.37	0.36	0.32	TAPPI T411 [34]
Air permeability (L/(m^2^·s))	137	106	243	ASTM D737 [35]
Gravimetric efficiency (@2000 Pa) (%)	99.9	99.8	99.9	ISO 5011 [36]
Pressure drop (@11.1 cm/s) (Pa)	162	209	91	ISO 5011 [36]
Stiffness (machine direction) (mN·m)	3.0	3.7	2.6	TAPPI T489 [37]

## References

[1] Chun Y., Kim J., Choi J.C., Boo K.O., Oh S.N., Lee M. (2001). Characteristic number size distribution of aerosol during Asian dust period in Korea. Atmos. Environ..

[2] Csavina J., Field J., Taylor M.P., Gao S., Landázuri A., Betterton E.A., Eduardo S.A.A. (2012). Review on the importance of metals and metalloids in atmospheric dust and aerosol from mining operations. Sci. Total Environ..

[3] Lakshminarayanan P.A., Nayak N.S. (2011). Wear in the heavy duty engine. Critical Component Wear in Heavy Duty Engines.

[4] Sherburn P.E. (1969). Air cleaner design—Present and future. SAE Tech. Pap..

[5] Treuhaft M.B. (1993). The use of radioactive tracer technology to measure engine ring wear in response to dust ingestion. SAE Tech. Pap..

[6] Jaroszczyk T., Petrik S., Donahue K. (2009). Recent development in heavy duty engine air filtration and the role of nanofiber filter media. J. Kones.

[7] Schilling A. (1972). Automobile Engine Lubrication.

[8] Wilcox M., Baldwin R., Garcia-Hernandez A., Brun K. (2010). Guideline for Gas Turbine Inlet Air Filtration Systems.

[9] Calculation Method for the Energy Use Related to Air Filters in General Ventilation Systems, Eurovent 4/21. https://eurovent.eu/?q=content/eurovent-421-2014-calculation-method-energy-use-related-air-filters-general-ventilation.

[10] Long J., Tang M., Liang Y., Hu J. (2018). Preparation of fibrillated cellulose nanofiber from lyocell fiber and its application in air filtration. Materials.

[11] Podgórski A., Bałazy A., Gradoń L. (2006). Application of nanofibers to improve the filtration efficiency of the most penetrating aerosol particles in fibrous filters. Chem. Eng. Sci..

[12] Zhang Q., Welch J., Park H., Wu C.Y., Sigmund W., Marijnissen J.C.M. (2010). Improvement in nanofiber filtration by multiple thin layers of nanofiber mats. J. Aerosol Sci..

[13] Hung C.H., Leung W.F. (2011). Filtration of nano-aerosol using nanofiber filter under low peclet number and transitional flow regime. Sep. Purif. Technol..

[14] Wang C., Otani Y. (2013). Removal of nanoparticles from gas streams by fibrous filters: A review. Ind. Eng. Chem. Res..

[15] He Z., Zhang X., Batchelor W. (2016). Cellulose nanofibre aerogel filter with tuneable pore structure for oil/water separation and recovery. RSC Adv..

[16] Si Y., Fu Q., Wang X., Zhu J., Yu J., Sun G., Ding B. (2015). Superelastic and superhydrophobic nanofiber-assembled cellular aerogels for effective separation of oil/water emulsions. ACS Nano.

[17] Esfahani H., Jose R., Ramakrishna S. (2017). Electrospun Ceramic Nanofiber Mats Today: Synthesis, Properties, and Applications. Materials.

[18] Yuan W., Fang G., Li Z., Chen Y., Tang Y. (2018). Using electrospinning-based carbon nanofiber webs for methanol crossover control in passive direct methanol fuel cells. Materials.

[19] Choi J., Yang B.J., Bae G.N., Jung J.H. (2015). Herbal extract incorporated nanofiber fabricated by an electrospinning technique and its application to antimicrobial air filtration. ACS Appl. Mater. Inter..

[20] Chang C.Y., Chang F.C. (2016). Development of electrospun lignin-based fibrous materials for filtration applications. BioResources.

[21] Barhate R.S., Ramakrishna S. (2007). Nanofibrous filtering media: Filtration problems and solutions from tiny materials. J. Membr. Sci..

[22] Saleem M., Gernot K. (2007). Effect of filtration velocity and dust concentration on cake formation and filter operation in a pilot scale jet pulsed bag filter. J. Hazard. Mater..

[23] Zhang S., Liu H., Yin X., Yu J., Ding B. (2016). Anti-deformed polyacrylonitrile/polysulfone composite membrane with binary structures for effective air filtration. ACS Appl. Mater. Interfaces.

[24] Wang N., Yang Y., Al-Deyab S.S., El-Newehy M., Yu J., Ding B. (2015). Ultra-light 3D nanofibre-nets binary structured nylon 6–polyacrylonitrile membranes for efficient filtration of fine particulate matter. J. Mater. Chem..

[25] Wang N., Si Y., Wang N., Sun G., El-Newehy M., Al-Deyab S.S., Ding B. (2014). Multilevel structured polyacrylonitrile/silica nanofibrous membranes for high-performance air filtration. Sep. Purif. Technol..

[26] Leung W.W.F., Hau C.W.Y., Choy H.F. (2018). Microfiber-nanofiber composite filter for high-efficiency and low pressure drop under nano-aerosol loading. Sep. Purif. Technol..

[27] Langner M., Greiner A. (2016). Wet-laid meets electrospinning: Nonwovens for filtration applications from short electrospun polymer nanofiber dispersions. Macromol. Rapid. Commun..

[28] Galka N., Saxena A. (2009). High efficiency air filtration: The growing impact of membranes. Filtr. Sep..

[29] Yao Y., Tang M., Yu T., Liang Y., Hu J. (2016). Filtration performance of dual-layer filter paper with fibrillated nanofibers. BioResources.

[30] Tang M., Hu J., Liang Y., Pui D.Y.H. (2017). Pressure drop, penetration and quality factor of filter paper containing nanofibers. Text. Res. J..

[31] Hu J., Liang Y., Wang Y., Zeng J. (2017). Self-cleaning air filtering material and preparation method therefor. U.S. Patent.

[32] Bernada P. (1996). Experimental study and modelling of binder migration during drying of a paper coating. Dry. Technol..

[33] (2013). Grammage of Paper and Paperboard (Weight per Unit Area).

[34] (2015). Thickness (Caliper of Paper, Paperboard, and Combined Board).

[35] (2018). Standard Test Method for Air Permeability of Textile Fabrics.

[36] ISO (2014). Inlet Air Cleaning Equipment for Internal Combustion Engines and Compressors—Performance Testing.

[37] (2015). Bending Resistance (Stiffness) of Paper and Paperboard (Taber-Type Tester in Basic Configuration).

[38] ISO (2016). Road Vehicles—Test Contaminants for Filter Evaluation—Part 1: Arizona Test Dust.

[39] Sun Z., Tang M., Song Q., Yu J., Liang Y., Hu J., Wang J. (2018). Filtration performance of air filter paper containing kapok fibers against oil aerosols. Cellulose.

[40] Leung W.W.F., Hung C.H. (2008). Investigation on pressure drop evolution of fibrous filter operating in aerodynamic slip regime under continuous loading of submicron aerosols. Sep. Purif. Technol..

[41] Leung W.W.F., Hung C.H., Yuen P.T. (2009). Experimental investigation on continuous filtration of sub-micron aerosol by filter composed of dual-layers including a nanofiber layer. Aerosol Sci. Technol..

[42] Tang M., Chen S.C., Chang D.Q., Xie X., Sun J., Pui D.Y.H. (2018). Filtration efficiency and loading characteristics of PM_2.5_ through composite filter media consisting of commercial HVAC electret media and nanofiber layer. Sep. Purif. Technol..

[43] Endo Y., Chen D.R., Pui D.Y.H. (1998). Effects of particle polydispersity and shape factor during dust cake loading on air filters. Powder Technol..

[44] Endo Y., Chen D.R., Pui D.Y.H. (2001). Air and water permeation resistance across dust cakes on filters—Effects of particle polydispersity and shape factor. Powder Technol..

